# The Diamagnetic Susceptibility of the Tubulin Dimer

**DOI:** 10.1155/2014/985082

**Published:** 2014-02-18

**Authors:** Wim Bras, James Torbet, Gregory P. Diakun, Geert L. J. A. Rikken, J. Fernando Diaz

**Affiliations:** ^1^Netherlands Organisation for Scientific Research, Dutch-Belgian Beamlines, European Synchrotron Radiation Facility, BP 220, 38043 Grenoble, France; ^2^Science and Technology Facility Council (STFC), Daresbury Laboratory, Cheshire WA4 4AD, UK; ^3^National Centre for Scientific Research (CNRS), National High Magnetic Field Laboratory, 143 Avenue de Rangueil, 31400 Toulouse, France; ^4^CIB Centro de Investigaciones Biológicas, Ramiro de Maeztu 9, 28040 Madrid, Spain

## Abstract

An approximate value of the diamagnetic anisotropy of the tubulin dimer, Δ*χ*_dimer_, has been determined assuming axial symmetry and that only the **α**-helices and **β**-sheets contribute to the anisotropy. Two approaches have been utilized: (a) using the value for the Δ*χ*_*α*_ for an **α**-helical peptide bond given by Pauling (1979) and (b) using the previously determined anisotropy of fibrinogen as a calibration standard. The Δ*χ*_dimer_ ≈ 4 × 10^−27^ JT^−2^ obtained from these measurements are similar to within 20%. Although Cotton-Mouton measurements alone cannot be used to estimate Δ*χ* directly, the value we measured, CM_dimer_ = (1.41 ± 0.03) × 10^−8^ T^−2^cm^2^mg^−1^, is consistent with the above estimate for Δ*χ*_dimer_. The method utilized for the determination of the tubulin dimer diamagnetic susceptibility is applicable to other proteins and macromolecular assemblies as well.

## 1. Introduction

Microtubules, MT, are elongated macromolecular structures composed of protofilaments, of tubulin dimers assembled in long hollow tubes. The tubulin dimer is kidney shaped and has an approximate molecular weight of 55 kDa. The assembled microtubule is on average composed of 13 protofilaments which make up a hollow tube with a maximum diameter of 24.6 ± 0.6 nm [1]. The length of this assembly *in vivo* is variable but can reach several microns.

The aligning effects of magnetic fields on macromolecular microtubules and other rigid fibrillar molecular assemblies are well known [1–3]. The driving force for magnetic orientation is the diamagnetic anisotropy of the tubulin subunits (dimers) combined with the large shape anisotropy and stiffness of the microtubules. Diamagnetic anisotropy originates from the anisotropic nature of chemical bonds and is more pronounced in resonance structures such as aromatic groups, peptide bonds, or double and triple carbon bonds [4].

A single peptide bond has a very weak diamagnetic anisotropy but when many of these bonds are linked together with a fixed and uniform orientation, as in *α*-helices and *β*-sheets, a relatively strong overall anisotropy can result [5].

The anisotropy of a single tubulin dimer is rather small, and so far the only effects of magnetic fields that have been observed are in concentrated assembled microtubule solutions [1, 3, 6] and microtubule containing structures [7–10]. Even for these systems, high magnetic fields (>8 Tesla) were required, although Vassilev et al. [2] reported high orientation in a significantly lower magnetic field for an individual microtubule in a diluted solution. An assessment of the value of the tubulin dimer anisotropy would be interesting from a fundamental point of view.

The molecular structure of the tubulin dimer has been elucidated by cryoelectron microscopy [11], and, in principle, this information could be used to determine the diamagnetic anisotropy by vectorially adding all the diamagnetically anisotropic components. However, assuming the aromatic amino acids have no overall preferred orientation, it should be possible to calculate the value of the diamagnetic anisotropy, to a good approximation, by considering only the contributions from the *α*-helices and *β*-sheets. Validation of this method by a direct cross correlation with experimentally obtained data is not possible since there is no method to measure the diamagnetic anisotropy directly. Therefore an indirect method has to be used.

In order to obtain experimental information about how a dilute solution of dimers responds to an applied magnetic field, Cotton-Mouton experiments can be performed [12]. For axial symmetric molecules and assuming a relatively low degree of orientation (<5% full alignment), the magnetically induced birefringence, Δ*n*, increases linearly with the square of the applied field, *B* ^2^. The Cotton-Mouton constant, *C*_CM_, is obtained from the slope; thus *C*_CM_ = Δ*n*/*λB* ^2^, where *λ* is the wavelength of the laser used in the birefringence experiments. The *C*_CM_ depends on the number of particles per unit volume and the product of their optical, Δ*α*, and diamagnetic, Δ*χ*, anisotropies; thus

(1) $$\begin{array}{c} C_{\text{CM}}=\frac{\Delta n}{\lambda B^{2}}\propto N\frac{\Delta \chi \Delta \alpha }{n_{0}15kT}\frac{1}{\lambda }, \end{array}$$

where *k* is the Boltzman constant and *T* is the absolute temperature of the solution.

Δ*χ* depends on the internal structure of the molecule while Δ*α* has potentially two components, one due to shape, form anisotropy, and the other, intrinsic anisotropy, due to internal structure.

Previous attempts to perform Cotton-Mouton experiments on tubulin dimers have been unsuccessful due to the low signal strength. Therefore, an improved protocol had to be designed with special care being taken to optimize the optical components of the equipment and stabilize the temperature of the solution. Experiments were performed on both dimers and double dimers, formed by the addition of Mg^2+^ ions.

## 2. Materials and Methods

### 2.1. Magnetic Birefringence and Cotton-Mouton Experiments

Magnetic birefringence [4] and Cotton-Mouton experiments, where the birefringence of the solution is measured as function of a steadily increasing magnetic field [12], were performed in the Grenoble High Magnetic Field Laboratory (Grenoble, France) using the optical equipment mounted on magnet M2. This magnet has a horizontal bore and can reach fields of upward to 17 Tesla [13]. For both experiments, the same optical equipment was used. In the Cotton-Mouton experiments the sample was maintained at constant temperature (4°C) and the magnetic field was ramped up and down at a constant *dB*/*dt* rate. All experiments were repeated a minimum of 6 times.

The effects that we were able to measure were rather weak and therefore care had to be taken to reduce the background contributions to the birefringence signal from the windows of the sample holder and from the solvent. The latter condition considerably reduces the possible choice of buffer solutions since one should avoid all buffers that cause optical Schlieren effects, that is, all buffer solutions with increased viscosity. In these experiments we found that only a tris(hydroxylmethyl)aminomethane (Tris) buffer did not introduce an unacceptable background.

The samples were placed in a thermostated holder and loaded in the magnet after which the magnetic field was ramped to 17 T.

### 2.2. Biochemistry

For the Cotton-Mouton experiments analytical ultracentrifugation was performed on the samples in order to determine the particle weight/size distribution. For these experiments, the tubulin is prepared in 20 mM Tris buffer with 0.1 mM guanosine triphosphate (GTP) and at a pH = 7.5. The samples were diluted to 2, 4, and 8 mg/mL. To one aliquot, 4 mM Mg^2+^ was added in order to induce the formation of oligomers and none to the other.

The sedimentation velocities were determined using a Beckman Optima XL-I ultracentrifuge (Beckman Coulter, Brea, USA) equipped with an interference optical detection system that allowed us to monitor the sedimentation of tubulin at high (0.1 millimolar) concentrations of nucleotide. Samples were studied at a speed of 40,000 rpm and 20°C by using an An50Ti eight-hole rotor and double-sector centerpieces (Beckman Coulter, USA). Differential sedimentation-coefficient distributions, *c*(*s*), were calculated by least-squares boundary modelling of sedimentation-velocity data, using the program SEDFIT [14].

The samples that were prepared without the addition of Mg^2+^ contained mainly (75 ± 5%) tubulin dimers, that is, the normal unit in which tubulin appears in vitro. A small amount of protein consisted of double or triple dimers (15 ± 5% and 7 ± 3%, resp.). This was independent of the protein concentration.

The samples to which Mg^2+^ was added had a more complex and concentration-dependent composition. At the lower concentration, 30 wt% of the material consisted of aggregates of several dimers. The remaining 70 wt% was composed of dimers and double dimers. At a concentration of 2 mg/mL the sample consisted of equal fractions of dimers and double dimers. The fraction of double dimers increased regularly as function of concentration such that at 8 mg/mL the fraction of material that was not assembled in larger aggregates (90 wt%) was solely composed of double dimers.

## 3. Results and Discussion

### 3.1. Cotton-Mouton Experiments on Dimers and Oligomers

**Figure 1:**
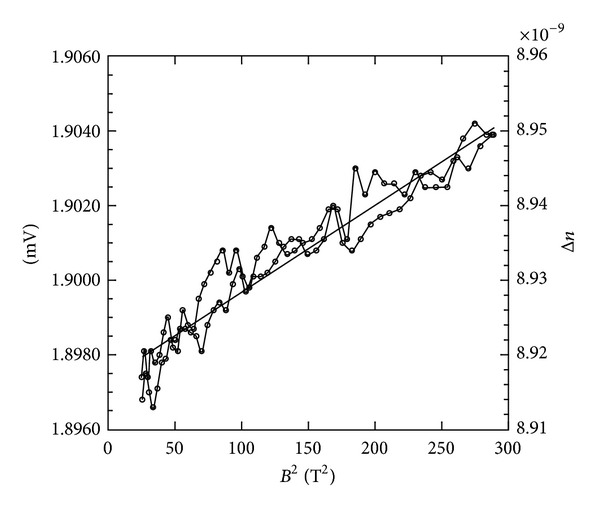
Representative example of Cotton-Mouton measurements on a dimer solution, 4 mg/mL, in Tris buffer. The magnetic field ramp rate was Δ*B*/Δ*T* = 50 (T min⁡^−1^). The results of the up and down sweep of the magnetic field are shown. A linear fit of the data (solid line) was used to obtain the Cotton-Mouton constant. Δ*n* is also expressed in mV to emphasize that these experiments are on the limit of sensitivity of the instrument. The statistical errors can be estimated from the variations of the data with respect to the linear fit.

In Figure 1, we show the Cotton-Mouton results obtained on a dimer solution of 4 mg/mL. This was the lowest concentration from which reproducible results could be obtained.

**Figure 2:**
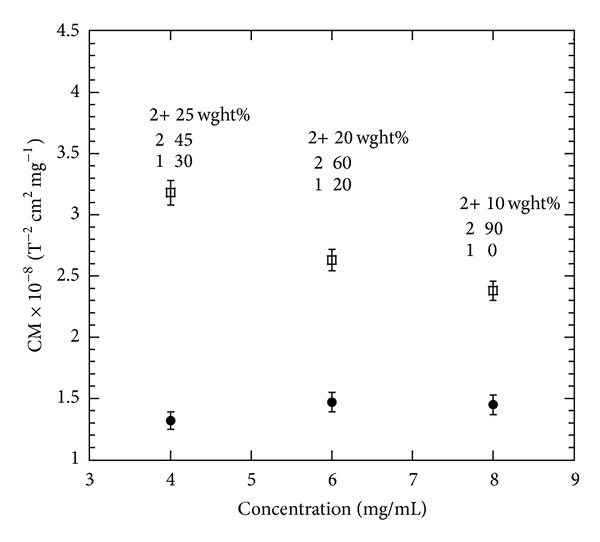
Cotton-Mouton constants, normalized to a concentration of 1 mg/mL, determined for both dimeric and double dimer samples as function of concentration. The estimated error margin is discussed in the text. The dimer solutions contain only a small, concentration independent fraction of larger aggregates. For the double dimer solutions, the composition is indicated in the figure. “2+” indicates the weight percentage of larger aggregates, “2” the weight percentage of double, dimers and “1” the amount of single dimers.

The dimer concentrations used were sufficiently dilute so that interdimer interactions could be ignored. This assumption is confirmed by the observation that the Cotton-Mouton constant normalised to the dimer concentration is independent of concentration (see Figure 2).

From these measurements we determined the value of the Cotton-Mouton constant for the dimer normalized to concentration to be CM_dimer_ = (1.41 ± 0.03) × 10^−8^ T^−2^cm^2^mg^−1^. This value is 11 times smaller than that of fibrinogen [15] but when normalized to molecular weight, *M*_*r*_, this difference is reduced by a third to 3.7 (*M*_*r*_ fibrinogen = 3 × *M*_*r*_ tubulin dimer). This comparison shows that tubulin dimers are significantly less anisotropic that fibrinogen molecules.

In order to have a cross check on the reliability of the data, oligomers were formed by adding MgCl_2_ and GTP. Mass determination via ultracentrifuge showed that the oligomers predominantly consist of double-dimers plus single dimers with a small proportion of larger oligomers (see Figure 2). The relative proportion of the different species was found to vary with concentration, and this is probably the reason for the nonlinear increase in the Cotton-Mouton constant as the concentration rises (see Figure 2).

The average Cotton-Mouton constant of the oligomer solution with the largest proportion of double dimers CM_dimer_ = (2.73 ± 0.03) × 10^−8^ T^−2^cm^2^mg^−1^ is about twice that obtained from single dimer solutions. This was expected as an approximate doubling of the Cotton-Mouton constant is the maximum that can be expected when dimers are transformed into double dimers. The anisotropy of double dimers depends on the relative orientation of the constituent single dimers and is maximal when the long axes of the single dimers are parallel to each other as in this configuration their anisotropies add together to a good approximation. Any other relative orientation of single dimers would result in lower anisotropy. It is expected that the long axes will indeed be nearly parallel since this is the normal arrangement when the dimers are incorporated in the protofilaments and the assembled microtubules. However, a small deviation angle between the long axes is feasible since the bond between dimers is not completely rigid.

Unfortunately, the magnetic fields available today are nowhere near strong enough to give rise to complete orientation. This limits further data interpretation since without the saturation value in birefringence it is not feasible to determine the optical anisotropy and hence the diamagnetic anisotropy from the CM constant [16]. One has to revert to comparisons with known materials.

### 3.2. Calculations of the Diamagnetic Moment

By idealizing the tubulin dimer as being axially symmetric, it is possible to obtain an approximate estimate of its diamagnetic anisotropy, Δ*χ*_dimer_, by summing the contributions from the *α*-helices and *β*-sheets. In this we assume that the other potential sources of diamagnetic anisotropy, principally the aromatic amino acids, have no net preferred orientation.

Firstly, each *α*-helix and *β*-pleated sheet was identified, the number of peptide bonds in each, (*N*_*α*_, *N*_*β*_), was totaled, and the angle (*θ*_*α*_, *θ*_*β*_) between the tubulin symmetry axis and the long axis of these secondary structural elements was estimated. The orientation factors (*f*_*α*_, *f*_*β*_) with (*f*_*α*,*β*_ = 1.5 × cos⁡^2^ *θ*_*α*,*β*_ − 0.5) could thus be obtained for each group. From this information Δ*χ*_dimer_ was estimated using the following equation adapted from Torbet and Maret [16]:

(2) $$\begin{array}{c} \Delta \chi_{\text{TD}}=\underset{i}{\sum }{\left(f_{\alpha }N_{\alpha }\Delta \chi_{\alpha }\right)}_{i}+{\left(f_{\beta }N_{\beta }\Delta \chi_{\beta }\right)}_{i}. \end{array}$$

Δ*χ*_*α*_ and Δ*χ*_*β*_ are the diamagnetic anisotropies of a single peptide bond in either an *α*-helical or a *β*-pleated sheet conformation. Due to the difference in conformation, Δ*χ*_*β*_ is only 25% of Δ*χ*_*α*_ (3) so *α*-helices are in general expected to make a greater contribution to the total Δ*χ*_dimer_ than *β*-pleated sheets and we can simplify (2) to

(3) $$\begin{array}{c} \Delta \chi_{\text{TD}}=\Delta \chi_{\alpha }\underset{i}{\sum }{\left(f_{\alpha }N_{\alpha }\right)}_{i}+0.25{\left(f_{\beta }N_{\beta }\right)}_{i}. \end{array}$$

In this way we estimate Δ*χ*_dimer_ = 83.5Δ*χ*_*α*_ with *β*-sheets contributing only 15% to the total anisotropy. Unfortunately the value of Δ*χ*_*α*_ is not accurately known. Two different estimates for Δ*χ*_*α*_ have been published, Pauling [17] gives 4.45 × 10^−29^ JT^−2^ while Worcester's [5] estimate is 60% greater. The former value is probably more reliable as it is closer to experimental estimates [18, 19] and is consistent with magnetic birefringence measurements made on two filamentous phages [16]. Thus, using Pauling's value we obtain Δ*χ*_dimer_ = 3.7 × 10^−27^ JT^−2^.

The value of Δ*χ*_dimer_ can also be estimated using fibrinogen for calibration as follows. The diamagnetic anisotropy can be calculated using the Cotton-Mouton constant and the birefringence at complete orientation or saturation orientation, Δ*n*_sat⁡_, using the relationship [16] Δ*χ* = 15*λkTC*_CM_/Δ*n*_sat⁡_. This equation cannot be exploited with tubulin as we do not have completely aligned samples. However, by putting the published values [15] for the Cotton-Mouton constant of fibrinogen and the saturation birefringence of fibrin into the latter equation we obtain 5 × 10^−26^ JT^−2^ for the Δ*χ* of a single fibrinogen molecule. Again assuming aromatic residues make no net contribution, this anisotropy is due to the axially aligned *α*-helices constituting about 30% of the molecule (i.e., 930 residues). The Δ*χ* of fibrinogen is thus equal to 930 × Δ*χ*_*α*_ as shown above. By comparison with fibrinogen, Δ*χ*_dimer_ = 4.5 × 10^−27^ JT^−2^ (i.e., 5 × 10^−26^ × 83.8/930 JT^−2^). This value is 20% larger than that calculated above using the Pauling value for Δ*χ*_*α*_.

The Δ*χ* of fibrinogen is 3.7 times larger than that of Δ*χ*_dimer_ normalized to molecular weight. As reported above the *C*_CM_ for fibrinogen is also 3.7 times larger than that of tubulin. This supports our estimate for Δ*χ*_dimer_; however, this conclusion assumes that the diamagnetic and optical anisotropies are linked by the same proportionality in fibrinogen and tubulin. This is probably approximately true for the intrinsic component of the optical anisotropy because, like the diamagnetic anisotropy, it depends on the anisotropic mobility of electrons in the molecule [20]. But the optical anisotropy can also have a form component which might be relatively different for fibrinogen and tubulin. While the Cotton-Mouton measurements are supportive of our estimate for Δ*χ*_dimer_, they do not constitute conclusive evidence.

Δ*χ*_dimer_ can be used to obtain an estimate of the minimum number of tubulin dimers, acting cooperatively, required to attain a highly oriented system. It is known [21] that for a diamagnetically anisotropic object to attain better than 80% maximum orientation in a magnetic field, *B*, then Δ*χB* ^2^ > 20*kT*. In a very strong magnetic field of 10 Tesla at 20°C the minimum number of tubulin dimers required, *N*_*d*_, is estimated to be in excess of 2 × 10^5^ (*M*_*r*_ > 2 × 10^10^ daltons) using the lower value for Δ*χ*_dimer_ calculated above. As diamagnetic anisotropy is additive to a good approximation, Δ*χ* = *N*_*d*_ × Δ*χ*_dimer_ for dimers arranged in parallel. Thus, if a single microtubule is undergoing orientation, in the absence of interaction with its neighbors and assuming a dimer length of 8 nm along the protofilament direction and an average of 13 dimer protofilaments in the tubulin wall, it would need to have a length in excess of (2 × 10^5^ × 8 nm)/13 ≈ 12 *μ*m, or alternatively a number of smaller microtubules orienting cooperatively could give rise to the same result. It should be pointed out that in concentrated solutions of rigid molecules like microtubules [22] The magnetic field might not be the only cause of alignment. Above a certain concentration, phase separation and subsequent formation of oriented domains could occur [23]. However, the directors of these domains will still display random orientation. The magnetic field will force these directors to become aligned with the magnetic field. Further research on this has been done but falls outside the scope of this paper.

## 4. Conclusions

We have been able to measure the Cotton-Mouton constant of tubulin dimers with a reasonable accuracy. The absence of a reliable value for the optical polarizability prevents a direct determination of the diamagnetic susceptibility but by comparing the Cotton-Mouton constants of the tubulin dimer with fibrinogen, for which the susceptibility is known, we can make a reasonable estimate. This estimate of the susceptibility corresponds well with the calculated contributions of the *α*-helices and *β*-sheets to the diamagnetic susceptibility. If the crystallographic structure is known these, calculations are relatively simple and can be carried out for other proteins as well. The cross correlations that can be made between the experimental results and the simple calculations validate the calculation method for the tubulin dimer and show that this method can be used as an initial assessment of the diamagnetic susceptibility of proteins for which no other data is available. The method that we have used to determine the tubulin dimer diamagnetic susceptibility can be used, for other, proteins and macromolecular assemblies for which this information is not readily available. This can become relevant in for instance medically applied Magnetic Resonance Imaging (MRI) where the applied fields keep increasing.
